# Complex Reconstruction of the Knee with a Free Vertical Rectus Abdominis Flap after Periprosthetic Soft Tissue Necrosis

**DOI:** 10.7759/cureus.3969

**Published:** 2019-01-28

**Authors:** David Perrault, Oscar J Manrique, Gene Lee, Antione L Carre, Daniel A Oakes, Alex K Wong

**Affiliations:** 1 Plastic Surgery, Keck School of Medicine of USC, Los Angeles, USA; 2 Plastic Surgery, Mayo Clinic, Rochester, USA; 3 Orthopaedics, Keck School of Medicine of USC, Los Angeles, USA

**Keywords:** free flap, periprosthetic infection, rectus abdominis, pedicled flap, knee reconstruction, total knee arthroplasty, gastrocnemius muscle flap, microsurgery, plastic surgery

## Abstract

Periprosthetic joint infection (PJI) is limb threatening and difficult to treat. Although a two-stage re-implantation is accepted as the standard of care for PJI, extensive debridement, numerous surgeries, or liquifactive necrosis from the infection can result in a tissue defect. With a large tissue defect, soft tissue coverage is required to protect the prosthesis, fill any dead space, and obtain a satisfactory wound closure. With defects too large for local or regional flap coverage, free tissue transfer is the best option. We present a case in which soft tissue coverage with both medial and lateral gastrocnemius muscle flaps was not sufficient; free tissue transfer was necessary for both wound closure and creation of an adequate soft tissue envelope for the future placement of a prosthesis. Regardless of the complicated surgical history and extensive soft tissue damage, limb function was restored and the patient regained his independence.

## Introduction

Wound complications after total knee arthroplasty (TKA) can be devastating. In particular, periprosthetic joint infection (PJI) is limb threatening and reportedly occurs in 1.8% of TKA cases [1]. These infections are notoriously difficult to treat; in one single center analysis of 3,270 TKAs, failure rates of incision and drainage (I&D) and two-stage resection and re-implantation procedures were 67% and 50%, respectively [2]. As reviewed by Rao et al., two-stage re-implantation is accepted as the standard of care for PJI [3]. Nevertheless, extensive debridement, numerous surgeries, or liquifactive necrosis from infection can occasionally result in a tissue defect. In the case of a large tissue defect, soft tissue coverage is required to protect the prosthesis, fill any dead space, and obtain a satisfactory wound closure.

We present a case of a severe chronic PJI that required multiple operations and, ultimately, free tissue transfer for wound closure. A brief overview of the decision making considerations when reconstructing a large tissue defect from periprosthetic soft tissue necrosis is also presented.

## Case presentation

**Figure 1:**
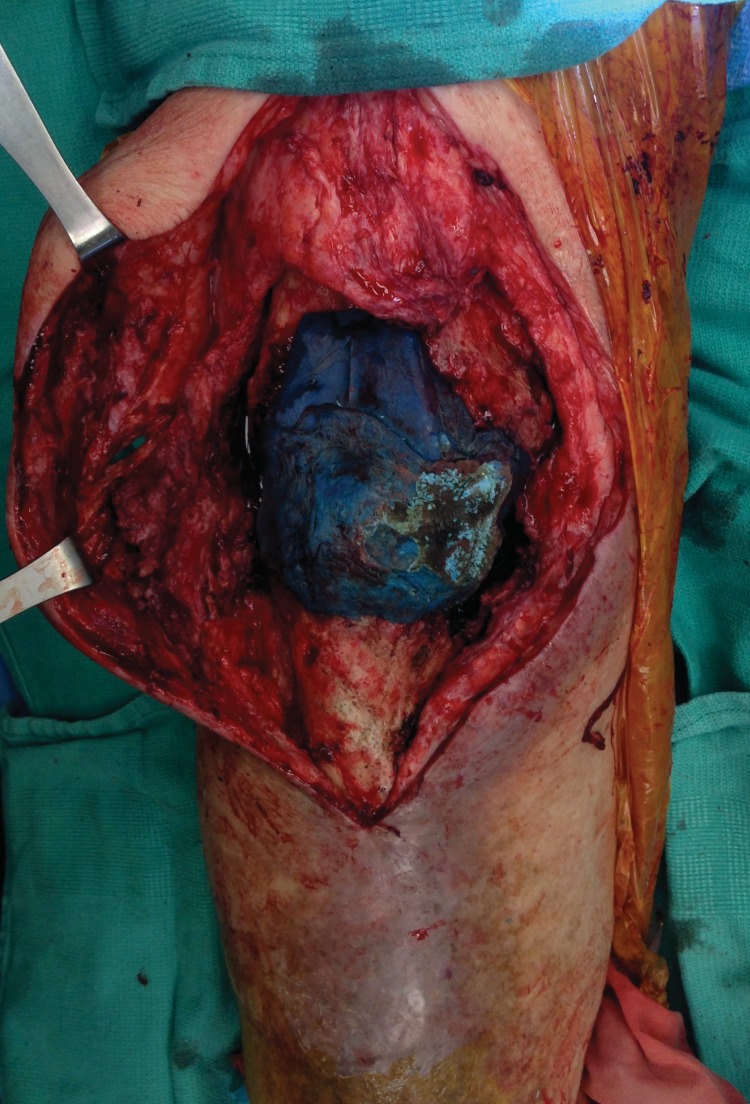
Intra-operative photograph of the right knee, showing the extensive soft tissue defect

A 56-year-old man with a history of ischemic cardiomyopathy, diabetes, hypertension, and 30 pack-years of smoking, presented to our care in April of 2014 for an acutely infected right TKA, originally done in 2004. He had previously undergone a failed two-stage resection and implant in 2008. Three weeks prior, he was admitted to an outside hospital for re-infection of his knee, where wound and blood cultures were positive for oxacillin sensitive Staphylococcus aureus. He underwent I&D on three occasions with an eventual placement of vacuum-assisted closure (VAC) device. The wound could not be cleared and he was referred to our care. Intra-operatively, purulent material surrounded the knee. The patellar tendon, medial retinaculum, and distal quadriceps had been completely eroded (Figure 1). Radical debridement with extensive skeletonization of the proximal tibia and distal femur was performed, including resection of the patella and removal of the infected TKA. A non-biodegradable drug delivery implant was placed. Medial and lateral gastrocnemius muscle flaps were rotated and covered 95% of the spacer. There remained a 6 x 6 cm area of exposed tibia devoid of periosteum and due to the patient’s medical co-morbidities, we did not feel that the patient was fit for a prolonged microsurgical free flap transfer. Therefore, the entire wound was covered with a matrix dermal regeneration template (Integra, Life Sciences, Plainsboro, NJ) and bolstered by a non-adherent dressing and VAC therapy sponge. The post-operative course was remarkable for bilateral pleural effusions secondary to heart failure and a small hematoma that formed over the wound on post-operative day five, which was evacuated at the bedside.

Three weeks later, the Integra was removed. Five weeks after, the Integra matrix was approximately 50% covered with granulation tissue and parts of the skin edges were necrotic. The patient was given the option of an above knee amputation or a free tissue transfer for wound closure as a skin graft would not have provided an adequate soft tissue envelope for a future joint prosthesis.

**Figure 2:**
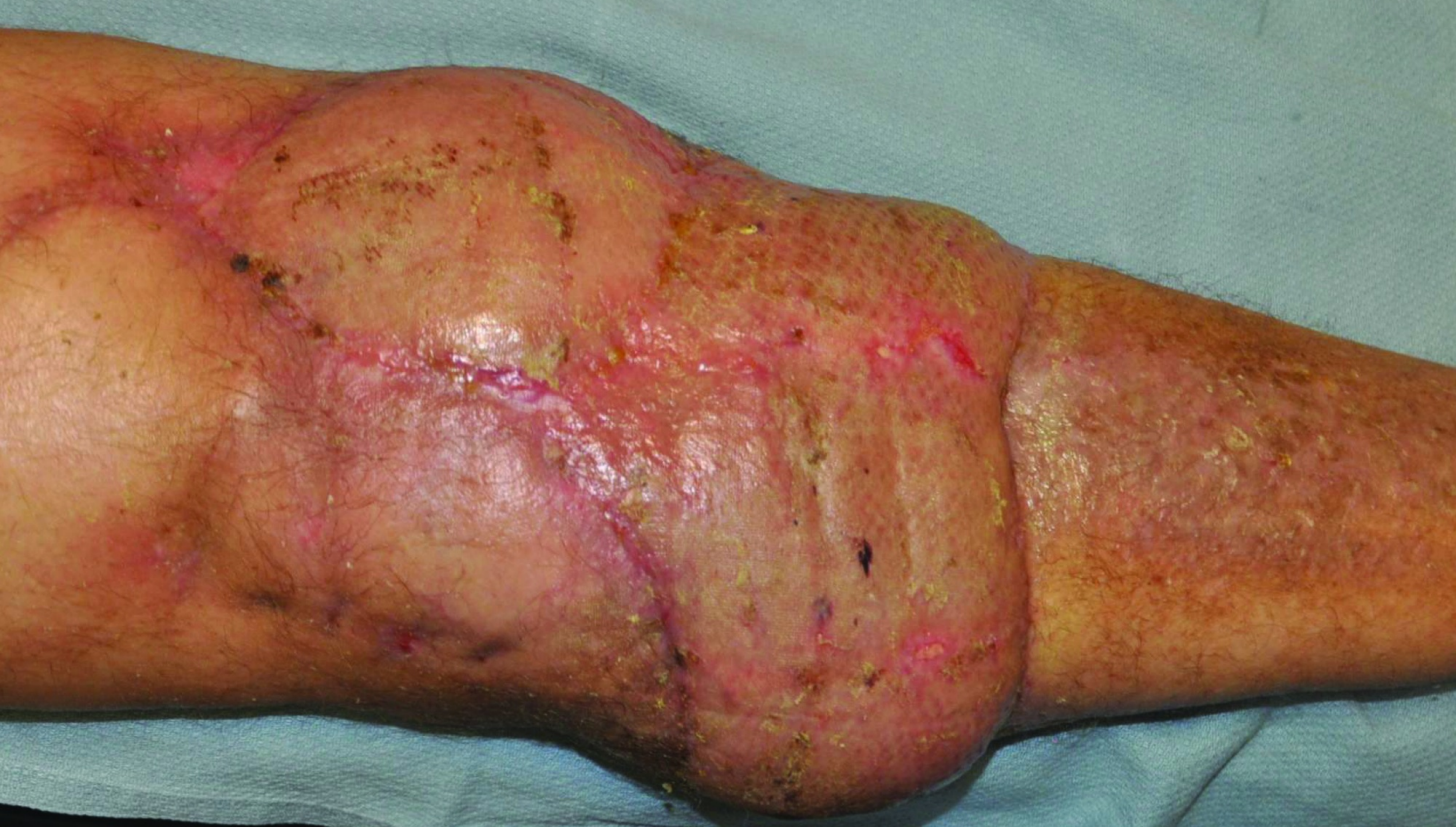
Post-operative photograph of the well-healed right knee

Eight weeks after, the wound bed was debrided and a vertical rectus abdominis free flap was transferred to the right knee, where the vascular pedicle was anastomosed to the posterior tibial artery in a side-to-end manner and two posterior tibial veins with venous couplers. A Cook-Swartz implantable Doppler was placed around the deeper of the two veins. A meshed split-thickness skin graft was fixed in place. Tissue specimens were positive for vancomycin-resistant Enterococcus and Candida tropicalis. Post-operative course was otherwise unremarkable. Eight months later, a TKA and extensor mechanism repair with an Achilles tendon allograft was performed with the aid of the plastic surgeon for flap elevation and complex closure of the wound. Eleven months after his initial presentation, the patient was satisfied with the outcome as he had regained his independence and could drive. His right knee demonstrated a passive range of motion from 0°-50° and active range of motion from 20°-60°. The incisions and donor graft site were well healed with no evidence of infection (Figure 2).

## Discussion

PJI can result in large soft tissue defects around the knee. The surgical options for wound closure around the knee follows the traditional reconstructive ladder [3]. In the presence of infection or with exposed hardware/vital structures, the options are elevated to flap coverage [4]. For small skin defects (<4 cm), local perforator flaps are a satisfactory option [5]. With larger defects, the use of a pedicled muscle or musculocutaneous flap is preferred [3]. As previously reviewed, there have been a number of case reports describing the use of local flaps for reconstruction of soft tissue defects around the knee [6]. The medial gastrocnemius muscle flap remains the workhorse method of reconstruction for soft tissue coverage of an exposed TKA [7]. However, with defects that are too large for local or regional flap coverage, free tissue transfer is necessary.

Free flap reconstruction of the knee is challenging because of the wide range of motion about the joint and the posterior location of the recipient vessels [8]. Despite this, free tissue transfer is possible for knee joint salvage [6,8]. Flap tissue type and recipient vessel selection are important considerations. Muscular flaps, versus fasciocutaneous flaps, provide a rich blood supply and optimize wound healing, delivery of humoral defenses, vascular drainage, and antibiotic delivery to the wound [5,9]. In this case, while there were several good options for donor flap site, including anterior lateral thigh, latissimus dorsi, rectus abdominus, the authors chose the latter due to patient donor site due to its reliable anatomy and lack of need for position change, both of which helped to reduces operative time in the patient with multiple co-morbid medical risk factors. With respect to recipient vessel selection, it is well accepted that it is vital to a successful flap transfer outcome [3,5,8,10]. Nevertheless, there is debate over which vessel is most optimal. We believe that a systematic and patient-specific approach to vessel selection should be used. The benefits and drawbacks of each vessel should be weighed. This information has been previously reviewed [5,10]. In this case, since the patient had palpable distal pulses at the ankle/foot, we did not elect to obtain pre-operative vascular imaging. In the authors' experience, the anterior or posterior tibial system is the first choice for recipient vessels due to proximity to the knee. As an alternative, the superficial femoral system proximal to the knee or geniculate system may be explored. With proper vessel selection, free tissue transfer can provide adequate soft tissue coverage around the knee allowing for restoration of motion.

## Conclusions

In this case, soft tissue coverage with both medial and lateral gastrocnemius muscle flaps was not sufficient; free tissue transfer was necessary for both wound closure and creation of an adequate soft tissue envelope for the future prosthesis. Despite the complicated surgical history and extensive soft tissue damage, limb function was restored.

## References

[1] Mahomed NN, Barrett J, Katz JN, Baron JA, Wright J, Losina E (2005). Epidemiology of total knee replacement in the United States Medicare population. J Bone Joint Surg Am.

[2] Wang KH, Yu SW, Iorio R, Marcantonio AJ, Kain MS (2015). Long term treatment results for deep infections of total knee arthroplasty. J Arthroplasty.

[3] Rao AJ, Kempton SJ, Erickson BJ, Levine BR, Rao VK (2016). Soft tissue reconstruction and flap coverage for revision total knee arthroplasty. J Arthroplasty.

[4] Nahabedian MY, Orlando JC, Delanois RE, Mont MA, Hungerford DS (1998). Salvage procedures for complex soft tissue defects of the knee. Clin Orthop Relat Res.

[5] Gravvanis A, Kyriakopoulos A, Kateros K, Tsoutsos D (2014). Flap reconstruction of the knee: a review of current concepts and a proposed algorithm. World J Orthop.

[6] Sun XG, Gong X, Song LS, Cui JL, Yu X, Liu B, Lu LJ (2016). Posterior thigh flap pedicled on the cutaneous vessels arising from the popliteo-posterior intermediate artery: a report of 5 cases. Ostomy Wound Manage.

[7] Wong AK, Pu LLQ, Sherman R (2013). Gastrocnemius flap. Reconstructive Surgery of the Lower Extremity.

[8] Louer CR, Garcia RM, Earle SA, Hollenbeck ST, Erdmann D, Levin LS (2015). Free flap reconstruction of the knee: an outcome study of 34 cases. Ann Plast Surg.

[9] Cetrulo CL Jr, Shiba T, Friel MT, Davis B, Buntic RF, Buncke GM, Brooks D (2008). Management of exposed total knee prostheses with microvascular tissue transfer. Microsurgery.

[10] Fang T, Zhang EW, Lineaweaver WC, Zhang F (2013). Recipient vessels in the free flap reconstruction around the knee. Ann Plast Surg.

